# Circuit quantum electrodynamics with dressed states of a superconducting artificial atom

**DOI:** 10.1038/s41598-022-26828-1

**Published:** 2022-12-24

**Authors:** Yu-Han Chang, Dmytro Dubyna, Wei-Chen Chien, Chien-Han Chen, Cen-Shawn Wu, Watson Kuo

**Affiliations:** 1 Department of Physics, National Chung Hsing University, Taichung, 402 Taiwan; 2 Department of Physics, National Changhua University of Education, Changhua, Taiwan; 3 Department of Electronic Engineering, Chung Yuan Christian University, Taoyuan, 320 Taiwan

**Keywords:** Electronics, photonics and device physics, Quantum physics

## Abstract

A dynamical control of the coupling strengths between dressed states and probe photon states is demonstrated with a transmon-like artificial atom coupled to two closely spaced resonant modes. When the atom is driven with one mode, the atom state and driving photon states form the so-called dressed states. Dressed states with sideband index up to 3 were prepared and probed via the strong coupling to the other resonant mode. Spectroscopy reveals that the coupling strengths are “dressed” and can be modulated by the power and sideband index of the driving. The transmission of the probe tone is modulated by the driving microwave amplitude with a Bessel behavior, displaying multi-photon process associated with the inter-atomic level transitions.

## Introduction

The development of superconducting quantum circuits has realized quantum optics at microwave frequencies in many aspects^1^. In particular, such a platform provides a great enhancement in the interaction between electromagnetic waves and the circuits, which are also called “artificial atoms”. Taking the advantage of strong coupling, a broad range of quantum optical phenomena, such as single atom spectroscopy^2^, dressed states^3^, amplification of light^4^, lasing^5^, quantum wave mixing^6,7^, Mollow triplets, Aulter-Townes splitting^8–10^, electromagnetically-induced transparency^11,12^, two-photon fluorescence^13^, fluorescence in squeezed vacuum^14^, emission of anti-bunched photons or photon multiplets^15,16^, and cooperative radiation in a topological waveguide^17^ have been demonstrated. In addition, wide tunability in superconducting quantum circuits as artificial atoms allows observation of various phenomena without restrictions in operation frequency.

Strongly driven two level system(TLS) may produce Landau-Zener (LZ) transition^18^ that can be demonstrated in various systems, such as an atom in an intensive laser field. LZ transition has been investigated in superconducting quantum circuits, such as Cooper-pair boxes(CPB)^3,19–21^, transmons^22^, and flux qubits^23,24^; as well as in quantum dots as spin qubits^25–28^ and charge qubits^29,30^. The large driving fields that can be achieved for these quantum circuits grant the phenomena observed to be significant. Under a strong drive, multi-photon processes occur when the TLS energy matches the energy of an integer number of photons. The population as a function of driving amplitude shows oscillatory behavior, a general signature of Landau-Zener-Stückelberg (LZS) interferometry^20,21,23,24^. The interference between these transitions becomes easy to access and control, that can be used for engineering quantum systems^31^.

For most atomic systems, such a multi-photon process is difficult to observe in open space due to the small atom-light interaction. Nevertheless, one may solve the problem by using a resonator to couple the driven artificial atom, in the scope of circuit quantum electrodynamics (QED)^32–38^. In particular, the strong coupling between an artificial atom and a resonator usually display a Jaynes–Cummings (JC) interaction^32,39,40^. When the coupling is dispersive, probing the resonator frequency implements a state measurement of high visibility for a two-level atom as a qubit^41–43^. Observations of the multi-photon transitions up to 5 photons has been reported by dispersively monitoring the transition^44^. Nonlinear response of the vacuum Rabi resonance is found when the atom and resonator are in resonance^45^. Schemes achieving ultrastrong coupling have also been shown^46,47^, revealing breakdown of the Jaynes–Cummings approximation^48^. In a driven system, qubit-resonator coupling in the dispersive regime present classical Bloch-Siegert shift^49^, while the Rabi oscillations in resonance with a resonator can introduce signal amplification^50^.

On one hand, circuit QED based on JC model can be used in quantum information processing, such as for coupling distant qubits^51^, 2-qubit operations^52,53^, entangling qubits^54^, preparation of the “cat state”^55^, photon storage^56^, and quantum memory^57^. On the other hand, there are many other extensions of the standard JC model, such as intensity-dependent coupling, multi-photon transitions, multi cavity modes^58^, damped JC model^59^, JC model with Kerr medium^60^, driven JC model^61^, the Tavis-Cummings model for many identical atoms^62^, and the Jaynes-Cummings-Hubbard model based on coupled resonators^63,64^.

**Figure 1:**
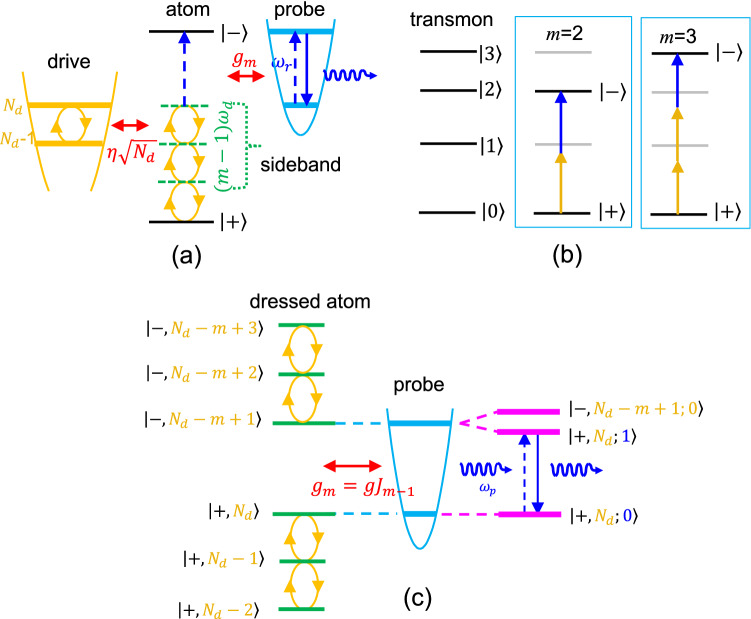
(**a**) The proposed scheme of circuit QED with a dressed artificial atom. A two-level atom is dressed with driving photon number $N_d$ with a bare coupling strength $\eta$ at a sideband transition to an excited atom level $|-\rangle$. Transitions in the dressed state is in resonance to a probe resonator with a modulated coupling strength, $g_m$. (**b**) The energy diagram of a transmon. When $m=2$ and 3, the TLS excited state is chosen to be $|2\rangle$ and $|3\rangle$, respectively. The orange and blue arrows illustrate the absorption of photons from the driving and probe resonators. (**c**) Dressed states are composite quantum states of $|\pm \rangle$ and $|N_d\rangle$. $g_m$ can be modulated by $N_d$ and sideband order $(m-1)$, and results in Rabi splitting of the spectrum. The probe channel is set at a frequency slightly detuned from the resonator frequency.

The implementation of circuit QED with an artificial atom is usually restricted to a fixed coupling strength, depending on the microwave structure of the quantum circuit. Inspired by the LZS interferometry of a driven two-level atom, we propose the concept of “dressed” coupling, controllable by the driving photon number, between a resonator and a dressed atom. To realize such a proposal, we consider a scheme that an artificial atom is coupled to two resonance modes as shown in Fig. 1a. One mode serves as the driving one for the preparation of the demanded dressed state with a driving photon number, $N_d$. It can be shown that the coupling of the dressed state to a probe resonator can be modulated by $N_d$ and sideband index *m*.

The desired atom system allowing multi-photon transitions can be implemented with transmons, of which hamiltonian reads $H_{{\text{atom}}}=\hbar \sum _{m=0} \omega _{m0} |m\rangle \langle m|$. The transmon energies are described by the anharmonicity $\alpha$ as $\omega _{m0}=m\omega _{10}-\frac{1}{2} m(m-1)\alpha$^65^. The ladder (or cascade) type atom allows resonances between the levels $|0\rangle$ and $|m\rangle$ with $m-$photon transitions at the condition of $\omega _{m0}=m\omega _d$, or $\omega _{10}-\omega _d=(m-1)\alpha /2$ with the photon frequency $\omega _d$^44^. Such a feature ensures the investigation of the “dressed” coupling of the sideband transition, close to these resonance condition when choosing the effective TLS as $|+\rangle =|0\rangle$(ground state) and $|-\rangle =|m\rangle$(excited state) as schematically shown in Fig. 1b for $m=2$ and 3 cases.

The essence of the scheme can be understood by using the model of a dressed TLS, and the drive is quantized as excitations of a harmonic oscillator^1^,

1 $$\begin{aligned} {H_{{\text{TLS}}}}=-\frac{1}{2}\hbar \omega _{a}\sigma _z+ {\hbar \omega _d}b^\dag b+\eta {\hbar \omega _d}(b^\dag +b)\sigma _k. \end{aligned}$$

$\hbar \omega _a$ is the two-level atom energy and $\omega _d$ is the driving frequency. $\eta$ can be interpreted as the dimensionless coupling energy between the TLS and driving photon field, of which *b* and $b^\dag$ are annihilation and creation operators. Pauli matrices are defined in the subspace expanded by the transmon state $|0\rangle$ and $|m\rangle$, and $k=x$ or *z* depending on the driving type. $N_d$ is related to the driving amplitude $\Omega _d\sim \eta \sqrt{\langle N_d\rangle }\omega _d$. While several works have pointed out that the X-driving has an exact numerical solution for the eigenstates^66^, the Z-driving model provides an analytical description of the dressed states $|\pm ,N_d\rangle =e^{\mp\eta \left( b^\dag -b\right) }|\pm \rangle |N_d\rangle$, allowing a simple analysis for the major features of the problem^3,19^.

The coupling between a dressed TLS and a probe resonator can be expressed by an additional JC interaction,

2 $$\begin{aligned} V_r= \hbar g\left( a^\dag +a \right) \sigma _x, \end{aligned}$$

where *a* and $a^\dag$ are annihilation and creation operators of the resonator photons. The coupling strength *g* usually depends on the microwave structure of the circuit. When there is a drive, the matrix elements of $V_r$ in the basis formed by dressed states can be written as:

3 $$\begin{aligned} \langle \pm ,N_d' |V_r |\mp,N_d\rangle = \hbar g\left( a^\dag +a \right) \langle \pm ,N_d' | \sigma _x |\mp,N_d\rangle \end{aligned}$$

As shown in Fig. 1c, when there is a resonance, $\omega _{a}-(m-1)\omega _d=\omega _r$, two composite states $|+,N_d;n_p=1\rangle$ and $|-,N_d-m+1;n_p=0\rangle$ are degenerate and mixed with a coupling strength, $g_m=g\times \langle +,N_d-m+1| \sigma _x |-,N_d\rangle$. Here $n_p$ is the photon number in the probe resonator. We note that this can be expressed as the matrix element of the displacement operator ${\mathscr {D}}(2\eta )=e^{2\eta \left( b^\dag -b\right) }$, taking a form of an exponential times an associated Laguerre polynomial. If the coupling is small but $\eta \sqrt{N_d}\sim 1$, the associated Laguerre polynomial can be approximated by the Bessel functions, namely $g_m=gJ_{m-1}(\nu )$ where $\nu =4\eta \sqrt{N_d}$^19^. The effective coupling, coined as “dressed” coupling modulated by driving photon number $N_d$ together with sideband index $(m-1)$ leads to possible dynamical control of circuit QED. Moreover, the coupling can be switched off at the zeros of Bessel functions.

In this paper, we report the dynamical control of couplings for dressed states to a resonator in various channels allowed by sideband transitions. We utilize a CPB/transmon-type artificial atom, which is strongly coupled to two different photon modes, supplying required *g* and $\eta$ in the proposed scheme. Avoided-crossing structures in spectrum assure the existence of the power-dependent coupling strengths. With a probe set slightly off-resonance $\delta _p=\omega _p-\omega _r$ to the resonator, we are able to observe resonance fluorescence when the sideband transition is in resonance to the probe mode. In particular, emission from the probe resonator can be modulated by the driving photon number, and can be completely turned off. The transmission amplitude follows the Bessel behavior as a function of driving amplitude with multi-photon processes up to $m=3$ and can be explained by the Z-driving model. In other words, our system effectively exhibits a 2 or 3-photon Jaynes-Cummings model depending on the selection of driving frequency and driving amplitude.

**Figure 2:**
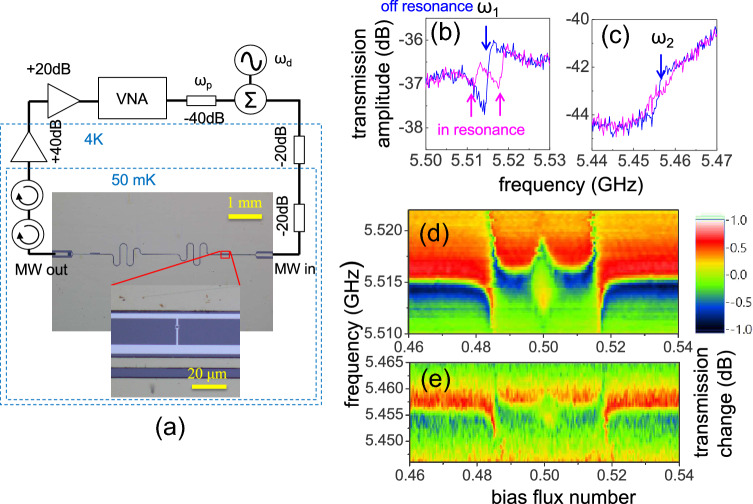
(**a**) The schematic of the 2-tone measurement. The transmon-like atom is placed in the slot of a transmission type resonator. (**b**) and (**c**) The microwave transmissions nearby the two resonances at 5.514 GHz (**b**) and 5.455 GHz (**c**). The curves are for the atom in resonance (red) and off-resonance (blue) with the 5.514 GHz mode. (**d**) and (**e**) The microwave transmission amplitude change as a function of bias flux number and frequency in the vicinity of 5.514 GHz (**d**) and the transmission amplitude change nearby 5.455 GHz (**e**). Both plots show the vacuum Rabi effect due to the coupling of the atom to the resonant modes.

## Methods

As illustrated in Fig. 2a, our sample is a transmon-like circuit coupled to a co-planar waveguide resonator^65^. The artificial atom and the resonator were made of Al and Nb, respectively on a Si substrate. We used finite element simulations (Ansys Q3D) to finely determine the coupling capacitances between electrodes in our design. The transmission-type resonator has an input and an output port with coupling capacitances of 5.98 fF and 83.3 fF, respectively. The atom is coupled to the signal line with a large capacitance 11.6 fF. The total capacitance of the atom, including those of junctions and that to the ground plane is estimated to be $\sim$27.5 fF, resulting in a charing energy $E_C/h$ of about 0.704 GHz. We will see later that it can be compared to the one obtained by transmon spectrum measurement. Such a value is larger than those in common transmon devices, because our design lacks interdigital electrodes, which can produce extra shunt capacitance across the junction. The Josephson coupling energy $E_J(0)/h$ in zero magnetic field is estimated as 150 GHz. A superconducting quantum interference device in the quantum circuit allows the tuning of transmon energies by biasing magnetic flux. The sample was measured in a dilution refrigerator at the base temperature below 40 mK. The transmission data was measured by using a commercial vector network analyzer and a spectrum analyzer.

## Results: atom spectroscopy

It turns out that the major resonator, which is expect to have a coupling strength of $\sim$80 MHz was not functioning normally and properly. Nevertheless, we found two high-Q resonant modes, which could display a strong coupling to the atom. Without flux-biasing, the atom is far detuned from both mode frequencies of $\omega _1/2\pi =$5.514 GHz and $\omega _2/2\pi =$5.455 GHz. Figure 2b,c respectively show the microwave transmission amplitude as a function of frequency in vicinity of the two modes. The blue curve is for the atom far off-resonance with both modes, while the red one is for the atom in-resonance with the higher mode. Figure 2d,e show the microwave transmission amplitude as a function of bias flux number and frequency in vicinity of $\omega _1$ and $\omega _2$, respectively. Avoided-crossing structures can be clearly seen when the atom and a mode is in resonance. Because the loss of the resonant mode is smaller than the coupling energy, we may resolve the splitting and estimate the mode-atom coupling energies, $2g_1/2\pi =$10 MHz and $2g_2/2\pi =$5 MHz. Though the origin of $\omega _2$ mode is unclear and the couplings are not as big as state-of-the-art values, the two modes could serve for our operation. This system is interesting because the frequencies of two resonant modes and atom transition can be tuned very close to each other. Important parameters of the device are summarized in Table 1.

**Table 1:** Important parameters of the circuit QED with artificial atom presented in unit of GHz. $\Delta _r=\omega _r-\omega _d$ and $\delta _p=\omega _p-\omega _r$.

Parameters	$E_J(0)/h$	$E_C/h$	$\alpha$	$\omega _1$	$\omega _2$	$g_1(=g)$	$g_2(=\omega _2\eta )$	$\Delta _r$	$\delta _p$
Values (GHz)	150	0.65	1.0	5.514	5.455	$5\times 10^{-3}$	$2.5\times 10^{-3}$	$5.9\times 10^{-2}$	$-10^{-3}$

**Figure 3:**
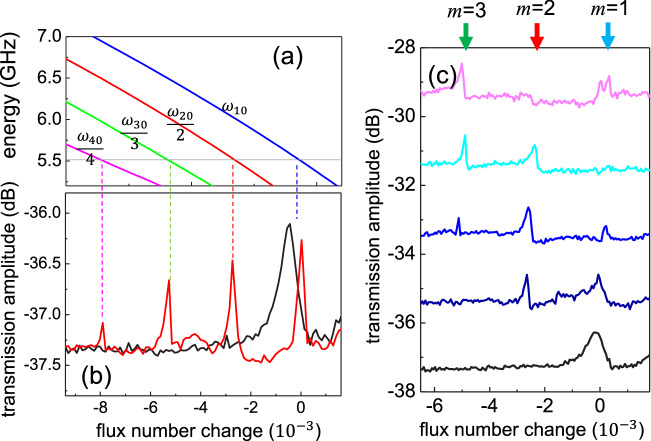
(**a**) The calculated transition energies $\omega _{m0}/m$ as a function of bias flux number in our atom. The grey horizontal line marks $\omega _p=5.5145$ GHz. (**b**) The transmission amplitude as a function of bias flux number(change) for different probe powers at 5.5145 GHz. At a lower power $P_p=-125$ dBm, only $|0\rangle$ to $|1\rangle$ transition was observed(black). At a higher power $P_p=-110$ dBm, $|0\rangle$ to $|m\rangle$ transitions with $m=$1, 2, 3 and 4 were observed (red). The peak positions agree well with the crossing points in (**a**). (**c**) The transmission of the probe microwaves at $\omega _p/2\pi =5.513$ GHz as a function of flux number change at various driving microwave powers at $\omega _d/2\pi =5.455$ GHz. The curves are vertically shifted for clarity. From the bottom to top, the driving power: off, $P_d=-108, -104.5, -102$ and − 100 dBm. In the absence of driving microwaves, the transmission shows a single resonance peak corresponding to transition energy $\omega _{10}=\omega _p$. For higher driving powers, the resonance peaks appear at flux number $=-2.6\times 10^{-3}$ and $-5.3\times 10^{-3}$, corresponding to $m=2$, and 3 in (**b**), respectively.

**Figure 4:**
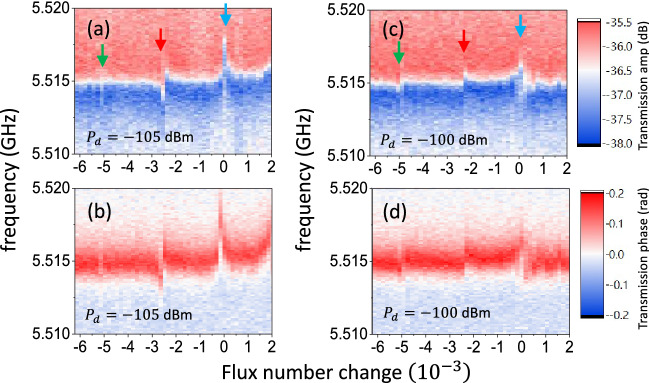
Avoided-crossing of dressed states. Transmission amplitude (**a**) and phase (**b**) as a function of flux number change at $P_d=-105$ dBm. Transmission amplitude (**c**) and phase (**d**) as a function of flux number change at $P_d=-100$ dBm. The blue, red and green arrows respectively indicate the resonance for $m=1$, 2 and 3 sidebands with the probe resonator.

When the probe frequency is detuned from $\omega _1/2\pi$ but not larger than $g_1$, we are able to observe the atom resonance due to the transition from ground $|0\rangle$ to the 1st excited state $|1\rangle$. This transition is shown in Fig. 3b as a resonance peak in the transmission amplitude at the bias flux number of 0.484 with the probe tone at a frequency of 5.5145 GHz and at the probe power $P_p=-125$ dBm(black curve). A strong probe power reveals very rich transmission structures featuring the resonant transition to a higher excited state with multiple photon absorption. The red curve in Fig. 3b for a high power $P_p=-110$ dBm shows three more resonance peaks located at an equal spacing of about $2.6\times 10^{-3}$ to the lower flux numbers.

As mentioned before, multi-photon resonances in transmon atom are expected to occur at the conditions $\omega _{10}-\omega _p=(m-1)\alpha /2$ with a probe frequency $\omega _p$. In our setup, the atom frequency $\omega _{10}$ is flux dependent with a conversion ratio of − 0.19 GHz per $10^{-3}$ flux number at the operation point. Given $\alpha = 1.0$ GHz, the resonance positions in flux number change can be simply written as $-2.6\times 10^{-3}(m-1)$, agreeing well with our observation. The charging energy can be determined as $E_C/h=0.65$ GHz by employing a CPB/transmon model presented in supplementary document. This $E_C$ value is consistent with what estimated from the device geometry and confirmed by other similar devices, which were tested using 2-tone measurement such as Aulter-Townes splitting^67^. The calculated photon energies $\omega _{m0}/m$ for *m*-photon transitions close to the probe frequency are illustrated in Fig. 3a for a confirmation of these transition at different bias flux numbers.

## Results: observation of dressed states

2-tone spectroscopy measurements were performed with a low probe power $-130$ dBm and a large driving microwave field at the frequency $\omega _d/2\pi =\omega _{2}/2\pi =$5.455 GHz. The probe frequency was chosen to be $\omega _{p}/2\pi =$5.513 GHz, about 1 MHz red detuned from $\omega _{1}/2\pi$. Again, such a small detuning allows us to clearly observe the atom spectrum shown as the black curve in Fig. 3c. In this operation, model parameters in Eqs. (1) and (2) can be assigned as $\eta =g_2/\omega _2$ and $g=g_1$. As the driving power increases, the resonance peak associated with $|0\rangle \rightarrow |1\rangle (m=1)$ transition begins to disappear. In the meanwhile, a resonance structure starts to build up at the position of $|0\rangle \rightarrow |2\rangle (m=2)$ transition in the 1-tone spectroscopy, followed by the appearance of a higher order $(m=3)$ one. However, unlike the monotonic behavior in 1-tone case, these peaks appear periodically in driving power. As such the resonance peaks associated with $m=1$ and $m=2$ transitions vanish completely and microwave transmission in these resonance channels are shut off at the powers of $P_d=-102$ dBm and $-100$ dBm, respectively.

To elucidate the origin of the resonance peaks associated with $m=2$ and 3, we conducted the spectroscopy measurement in vicinity of $\omega _1$. Figure 4a,b respectively illustrate the transmission amplitude and phase at $P_d=-105$ dBm in the flux bias region displaying $m=1$ to 3 channels. In addition to the avoided crossing for $m=1$ indicated by the blue arrow, one can clearly see the onset of avoided-crossing for $m=2$(red arrow). At an elevated $P_d=-100$ dBm, avoided-crossing can be found for $m=3$ (green arrow) in both transmission amplitude and phase plots as illustrated in Fig. 4c,d, respectively. The occurrence of avoided-crossing structures for $m=2$ and $m=3$ provide the evidence of the strong coupling between the probe resonator and dressed states attributed to the pronounced interaction of the bare atom and driving microwaves. The resonance conditions are given by $\omega _{m0}=(m-1)\omega _d+\omega _r=m\omega _d+\Delta _r\sim m\omega _d$, closed to that for aforementioned *m*-photon transition. Here $\Delta _r=\omega _r-\omega _d\ll \omega _d$.

**Figure 5:**
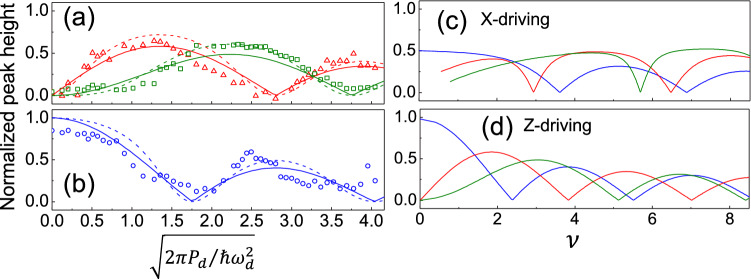
(**a**) and (**b**) Normalized peak height as a function of normalized driving microwave amplitude for $m=1$ (blue), 2 (red) and 3 (green) processes. All data sets are normalized by identical parameters. The solid curves are $|J_{m-1}|$ while the dash curves show the results by Eq. (5). (**c**) and (**d**) The calculated transmission amplitudes as a function of driving amplitude $\nu$ for X-driving model (**c**) and Z-driving model (**d**).

To investigate how the resonance structures are modulated by the driving power, we plot the peak heights in Fig. 3c against the normalized driving amplitude $\sqrt{2\pi P_d/\hbar \omega _d^2}$ as illustrated in Fig. 5a,b. The curve for $m=1$ channel reaches zero at the normalized driving amplitude of 1.75. Zeros are 2.8 for $m=2$ and 3.4 for $m=3$ channels, respectively. Again, the modulated resonance transmission of all channels suggest that the coupling strengths are modulated by driving power in an oscillatory manner.

## Discussion

Here, we provide a quantitative analysis of the oscillatory behavior in the transmission height by noting two practical points: finite probe photon number $n_p$, and the selection of $|-\rangle =|m\rangle$. Although the probe tone is weak, we cannot overlook the large energy conversion from an intensive driving to the probe resonator. Therefore, we may consider the situation that the resonator state no longer can be described by the photon number state, but a coherent state. Indeed, when the photon generating rate and the loss rate in a resonator are balanced, the resonator state is better described by a coherent state. For a specified coherent state $|\mu \rangle$, there is a most probable photon number state $|n_p=|\mu |^2\rangle$ and a spread of $n_p, \delta n_p \sim |\mu |$. The expanded quantum states(composited of dressed states and $n_p$ states) are noted as $|\pm , N_d; n_p\rangle$ with energy of $\mp\omega _a+N_d\omega _d+n_p\omega _r$.

We ever mentioned that when the transmon is biased such that its energy level spacing between ground state and *m*th excited state satisifying $\omega _{m0}=\omega _a= (m-1)\omega _d+\omega _r$, two expanded states $|+,N;n_p\rangle$ and $|-,N_d-m+1;n_p-1\rangle$ become degenerate and mixed with a coupling strength, $g\sqrt{n_p} \langle +,N_d-m+1| \sigma _x |-,N_d\rangle =g\sqrt{n_p}J_{m-1}(\nu )$. In vicinity of the degeneracy, the quantum system can be described by a 2-state subspace,

$$\begin{aligned} {H({n_p})}=\hbar {\omega _{0}}+\hbar \left( \begin{array}{cc} \Delta _r &{} g\sqrt{n_p}J_{m-1} \\ g\sqrt{n_p}J_{m-1} &{} \Delta _m \end{array} \right) . \end{aligned}$$

Here $\Delta _m=\omega _{m0}-m\omega _d$ is the detuning for *m*-photon transition and $\omega _0= N_d\omega _d+n_p\omega _r-(\omega _{m0}/2)$. In our case, $\Delta _r$ is small so that the resonance conditions are close to $\Delta _m=0$. When there is a resonance $\Delta _m\sim \Delta _r$, the lower energy eigenstate is given by $|\psi _0; n_p\rangle \sim \left( |+, N_d; n_p\rangle +|-, N_d-m+1; n_p-1\rangle \right) /{\sqrt{2}}$, which is the state we intended to probe.

According to the linear response theory, the transmission of the probe microwave is given by the matrix element, $T\propto \langle \psi _0; n_p| D|\psi _0;n_p \rangle$, in which destruction operator $D=\sigma _-$. Combining the expression of $|\psi _0;n_p \rangle$, one readily gets

4 $$\begin{aligned} T \propto \frac{1}{2} \langle +,N_d-m+1| \sigma _-|-, N_d\rangle = \frac{1}{2}J_{m-1}(4 \eta \sqrt{N_d}). \end{aligned}$$

The expression is also valid for a coherent state $|\mu \rangle$ when $\mu$ is large.

In the case of X-driving, the dressed state energy is $E(\pm ,N_d)/\hbar =N_d\omega _d\pm \frac{1}{2}\omega _a J_0(\nu )+O(\eta ^2)$^66^. Regardless how large is the atom level spacing, the two expanded states in resonance would be $|+, N_d; n_p\rangle$ and $|+, N_d+1; n_p-1\rangle$ with the coupling strength of $g\sqrt{n_p}\times \langle +, N_d+1|\sigma _x |+, N_d\rangle$. The lower mixed state is $|\psi _0; n_p\rangle \sim \left( |+, N_d; n_p\rangle +|+, N_d+1; n_p-1\rangle \right) /{\sqrt{2}},$ which results in a transmission,

$$\begin{aligned} T\propto \langle \psi _0;n_p| \sigma _- |\psi _0;n_p \rangle \simeq \frac{1}{2}\langle +, N_d+1|\sigma _-| +, N_d\rangle . \end{aligned}$$

Though the integrability and the route to exact solution of the X-driving model has been shown^66^, the matrix elements were calculated numerically when $\Delta _m\sim 0$ and illustrated in Fig. 5c, allowing a comparison to the Z-driving case shown in Fig. 5d.

The experimental |*T*| curves scaled with a single parameter($\sqrt{2\pi P_d/\hbar \omega _d^2}=\nu /1.4$) in the driving amplitude are in high accordance with the Bessel functions $|J_{m-1}(\nu )|$ predicted in Z-driving case presented by the solid curves in Fig. 5a,b. Provided $\nu =4\eta \sqrt{N_d}$ and $N_d={P_d}/{\gamma \hbar \omega _d}={2\pi P_d}{Q}/{\hbar \omega _d^2}$, the scaling factor is $4\eta \sqrt{Q}$, in which $Q=1/\gamma$ is the quality factor of the driving resonator. By knowing $Q\sim 3000$ obtained from transmission data and the scaling factor 1.4, we can estimate dimensionless coupling strength $\eta =6\times {10}^{-3}$, which is close to another estimation $g_2/\omega _2=0.5\times {10}^{-3}$.

Above results neglect the finite probe detuning, $\delta _p=\omega _p-\omega _r$ and decay rate $\gamma$. An analytical correction by using 2-level approximation reads

5 $$\begin{aligned} |T|\propto \frac{g^2J_{m-1}^2+\gamma ^2 }{\delta _p^2+g^2J_{m-1}^2+\gamma ^2}. \end{aligned}$$

The detailed derivation is presented in supplementary document. When $g\sim \delta _p \gg \gamma$, the result is consistent with Eq.(4). As an example, |*T*| with $g/\delta _p=2$ and $\gamma =0$ are shown as dash curves in Fig. 5a,b. There is slight change in peak height but zeros remain the same as those in Bessel dependences.

We would like to note that in the category of CPBs and transmons, the dominant electric dipolar interaction results in an interaction hamiltonian that can be described by the charge number operator *n*^68^. It has been pointed out that for a CPB in a 2-level approximation, the effective interaction is $\sigma _z(\sigma _x)$-type when the gate charge $n_g$ is close to 0(0.5), depending on the analogy to the application of an ac *z*(*x*) field to the spin magnetic resonance^68,69^. When $E_J/E_C> 10$, the circuit becomes less $n_g$-sensitive and $\sigma _x$-type interaction becomes dominant^65^. The power dependence in accordance with the Z-driving model is probably due to the low $E_J/E_C$ value, ranging from 11.5 to 15.5 at the operation point, where the atom frequency $\omega _{10}$ is varied from 5.5 to 6.5 GHz. As such, the driving from the resonator $\omega _2$ may not be X-driving as one will expect for ideal transmons. We expect that further investigations on similar quantum circuits with lower $E_J/E_C$ values could clarify this point.

## Conclusion

In conclusion, we have studied the microwave dressed states of a transmon-like atom coupled to a resonator. Avoided-crossing structures for sideband resonances are observed in spectrum, confirming the existence of the power-dependent coupling strengths. With a probe set slightly off-resonance to the resonator, we are able to observe resonance fluorescence when the sideband transition is in resonance to the probe mode. Emission from the probe resonator can be modulated by the driving photon number, following the similar power dependence in “dressed” coupling strength. The transmission amplitude as a function of the driving microwave amplitude via *m*-photon channel obeys the Bessel function $|J_{m-1}|$. At the zeros of Bessel functions, the output probe microwaves are completely suppressed, leading to the controlled darkening of these resonance channels. The output of probe photons also follows *m*-photon process and can find its application for a tunable source of correlated *m* photons.

## Supplementary Information

Supplementary Information.

## Data Availability

The datasets used and analysed during the current study are available from the corresponding author on reasonable request.
