# Supplementary material for: Circuit quantum electrodynamics with dressed states of a superconducting artificial atom
Source: Sci Rep. 2022 Dec 24;12:22308. doi: 10.1038/s41598-022-26828-1 (PMC9789979; doi:10.1038/s41598-022-26828-1)
Supplement: Supplementary file 1 — Supplementary Information. [file 41598_2022_26828_MOESM1_ESM.pdf]

supplementary documents on Circuit Quantum Electrodynamics with Dressed States of a Superconducting Artificial Atom

Yu-Han Chang,[1] Dmytro Dubyna,[1] Wei-Chen Chien,[1] Chien-Han Chen,[2] Cen-Shawn Wu,[2] and Watson Kuo[1,3]
[1]*Department of Physics, National Chung Hsing University, Taichung 402, Taiwan*
[2]*Department of Physics, National Changhua University of Education, Changhua, Taiwan*
[3]*email: wkuo@phys.nchu.edu.tw*

I. NUMERICAL CALCULATION

Without loss of important features, here we first provide a picture, in which the driving microwave is treated as a classical one. The hamiltonian for a cascade type atom is written in the following form:

$$H = H_a + H_d \;=\; \sum_{m=1} \hbar\omega_{m0}|m\rangle\langle m| + \sum_{m=0} \mu_{m+1,m}\mathcal{E}(e^{-i\omega_d t} + e^{i\omega_d t})|m+1\rangle\langle m| + h.c.$$

$m = \{0, 1, \cdots\}$ is the level index and $\omega_{m',m}$ is the level spacing between $|m\rangle$ and $|m'\rangle$. $\mathcal{E}$ and ω_d are the amplitude and angular frequency of the driving electric field. Here we consider the selection rule of the transmon that the matrix elements for electric dipole moment $\mu_{m',m}$ are zero if m and m' are both even or odd. For a transmon, the energies are $\omega_{m0} = m\omega_{10} - \frac{1}{2}m(m-1)\alpha$, described by anharmonicity α.

The behavior of the driven multi-level transmon can be understood using the rotational wave approximation and the definition of Rabi frequencies $\Omega_{m+1,m} = \sqrt{m+1}\Omega_d = \mu_{m+1,m}\mathcal{E}/\hbar$.

$$H_{\text{eff}} = \hbar\Delta_m|m\rangle\langle m| + \frac{\sqrt{m}}{2}\hbar\Omega_d|m-1\rangle\langle m| + h.c.$$

Diagonal elements for $m > 1$ are defined as $\Delta_m = m\Delta_1 - \frac{1}{2}m(m-1)\alpha$, in which detuning parameter is $\Delta_1 = \omega_{10} - \omega_d$. Such a system may have many resonances conditions that $\Delta_1 = (2m_0 + m - 1)\,\alpha/2$ between the levels $|m_0\rangle$ and $|m_0 + m\rangle$. Therefore, we might be able to observe the level splitting when Δ/α is half integers. Because of the selection rule of the transmon, the transition between $|m_0\rangle$ and $|m_0 + m\rangle$ can only be probed by m-photon processes. However, when the system is kept at the ground states $|m_0 = 0\rangle$, except $\Delta_1 = 0$, these rich resonances can only probed using multi-photon process when the probe is intense. In a two-tone measurement, in addition to the ordinary resonance transmission for $\omega = \omega_{10}$, other resonances can be assisted by the m-photon process allowed by the driving such that $\omega + (m-1)\omega_d = \omega_{m0}$, or $\omega - \omega_d = \Delta_m$. Such an effect will be observable when control is intense.

Here we proposed a way to probe these resonances by using a resonator to couple the driven transmon. The transmission spectrum would show the resonances we expected for the driven transmon using a weak probe. To understand the mechanism, let's consider the Jaynes-Cummings model describing the interaction between the readout resonator and the cascade type atom:

$$H = \hbar\omega_r a^\dagger a + H_{\text{atom}} + g\sum_{m=0}(a|m+1\rangle\langle m| + h.c.)$$

Here a and $a^\dagger$ are anihilation and creation operators of the resonator photons, while g is the coupling strength. It should clearly to find that the driven atom and resonator will have resonances at $\omega_r - \omega_d = \Delta_m$, in which $m = 1, 2\cdots$. By defining new detuning parameters $\Delta_r = \omega_r - \omega_d$, one can rewrite the resonances conditions to be

$$\Delta_1 = \frac{m-1}{2}\alpha + \frac{1}{m}\Delta_r. \tag{1}$$

When $m = 1$, it recover the ordinary atom-resonator resonance condition, $\delta = \omega_{10} - \omega_r = 0$.

In our system, the probing photons directly interact with the resonator, which also emit photons to the output terminal as schematically shown in SFig. 1(a) . With the approach by Kockum et al.[1], the transmission coefficient of the probe photon can be deduced by the susceptibility, which is given by

$$\chi(\omega) = i\int_0^\infty dt e^{i\omega t}\,\langle[\Sigma_-(t), \Sigma_p(0)]\rangle,$$

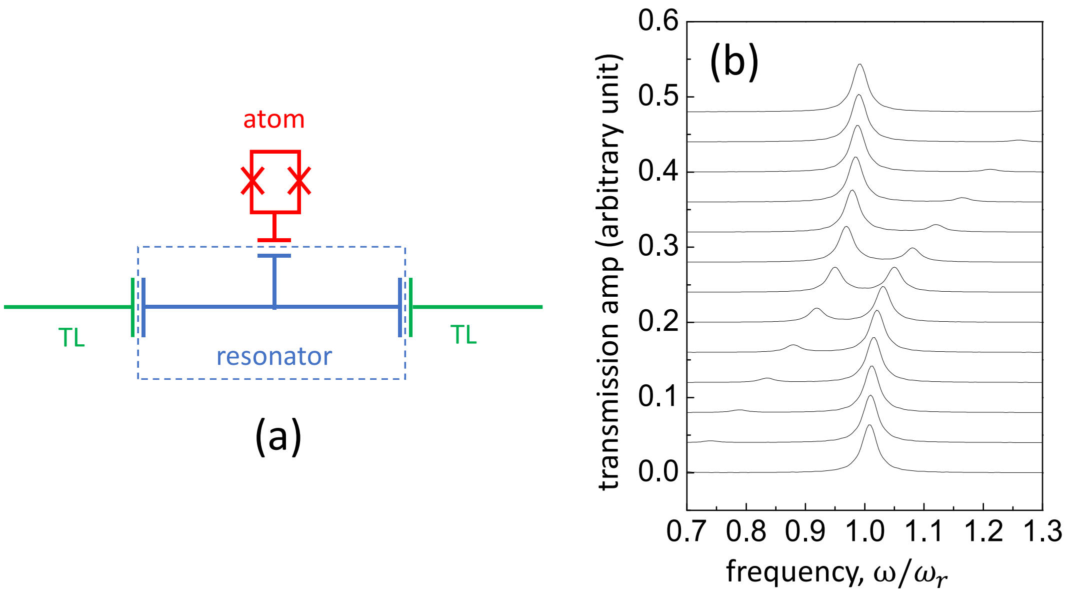

FIG. 1: (a) The structure of a transmon artificial atom coupled to a transmission type resonator, which coupled to two transmission lines(TL) for probing the resonances. (b) The calculated transmission amplitude of the TL at various atom detuning $\delta = \omega_{10} - \omega_r$ from $-0.3\omega_r$ to $0.3\omega_r$. It clearly displays the avoided crossing at $\delta = 0$. The coupling strength of the atom and resonator is assumed to be $g = 0.05\omega_r$, Losses for atom and resonators are assumed to be $\gamma = \Gamma = 0.01\omega_r$.

where $\Sigma_- = \sum_N \sqrt{N} \left|N-1\right\rangle \left\langle N\right|$ and $\Sigma_p = \Sigma_- + \Sigma_-^\dagger$ for resonator states $|N\rangle$. Quantum regression theorem states that the ensemble average of the time-correlation of two operators can be calculated by $\langle \Sigma_p(0)\,\Sigma_-(t)\rangle = Tr\left(\rho_p(t)\,\Sigma_-(0)\right)$, where $\rho_p(t)$ is the time evolution of the system density matrix with an initial condition of $\rho_p(0) = \rho_s \Sigma_p(0)$.[2] ρ_s is the density matrix for the steady state, which will be assumed to be ground state when the probe light is weak. The density matrix should follow the time-evolution equation of

$$\frac{d\rho}{dt} = -\frac{i}{\hbar}[H,\rho] + \sum \gamma \left(A\rho A^\dagger - \frac{1}{2}A^\dagger A\rho - \frac{1}{2}\rho A^\dagger A \right).$$

Operator A is collapse operator. For the resonator, $A = a$, while for the atom, $A = \sqrt{m+1}|m\rangle\langle m+1|$ and $\sum_m m|m\rangle\langle m|$ for the diagonal decay effect and dephasing effect, respectively. [1] The calculations can be undertaken numerically with the $N_{\max} \times m_{\max}$ dimensional Hilbert space, which is constructed by $|i\rangle = |N\rangle \bigotimes |m\rangle$. The possible multi-photon processes will be limited by the $m_{\max}$ used in the calculation.

To check whether the calculation scheme can provide the transmission data for our need, we firstly calculate the transmission amplitude for the standard c-QED problem with $N_{\max} = m_{\max} = 3$ and $g = 0.05\omega_r$ as shown in SFig. 1(b). The loss for the resonator and atom are assumed to be $\Gamma = \gamma = 0.01\omega_r$. In this strong coupling regime, $g > \Gamma, \gamma$, one can clear see that vacuum Rabi splitting occurs when $\delta = 0$ with the amount of $2g = 0.1\omega_r$. As the atom frequency detuned from ω_r, we can see its resonance becomes weaker and follows the analytical result

$$T_{\text{resonance}} \propto \left(1 - \frac{\delta}{\sqrt{\delta^2 + 4g^2}}\right).$$

The results for atom under driving are presented in SFig. 2. In the plot, $\alpha = 10^{-2}\omega_r$, $g = 5 \times 10^{-3}\omega_r$, $\Gamma = \gamma = 5 \times 10^{-4}\omega_r$ are used. These values are close to those in our experiment. The detuning factor for resonator $\Delta_r = -10^{-2}\omega_r$, and driving amplitude $\Omega_d = 1.5 \times 10^{-2}\omega_r$ and $\Omega_d = 2.5 \times 10^{-2}\omega_r$ are respectively assumed in SFig. 2 (a) and (b). In addition to the resonance due to the ω_{10} transition, one can also see the $m = 2$ and 3 cases. These multi-photon processes come across with ω_r at the conditions given by SEq. (1), and would cause much greater change in the transmission amplitude.

It is interesting to investigate how the resonator frequency change by the dressed atom, in particular when the conditions given by SEq.(1) are met. As such we conducted the calculations using $N_{\max} = 5$ and $m_{\max} = 4$ at $\delta/\omega_r = 3.5 \times 10^{-2}$, corresponding to the condition given by SEq. (2) with $m = 2$. As shown in SFig. 3(a), there is

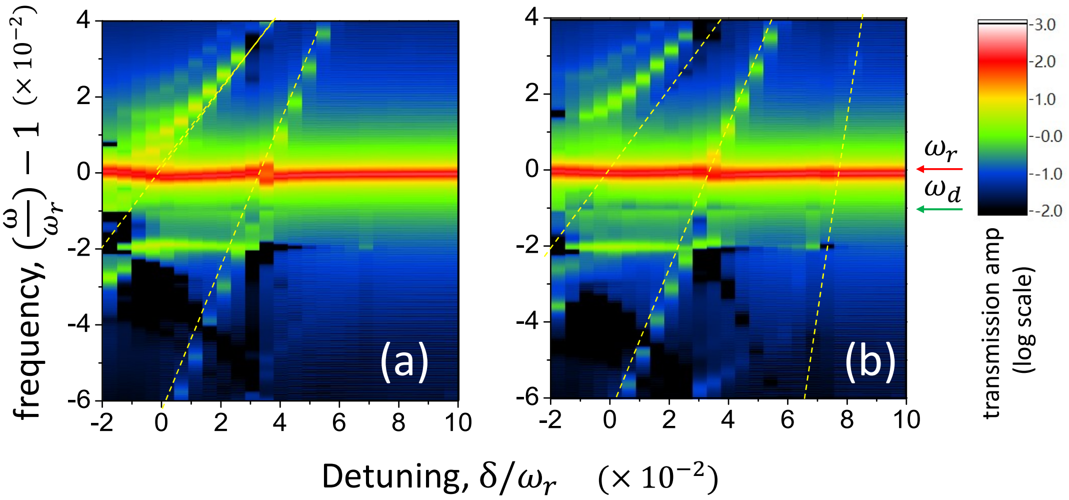

FIG. 2: (a)(b) The log-scale plot for transmission amplitude at different detuning δ/ω_r at the driving amplitude $\Omega_d = 1.5 \times 10^{-2}\omega_r$(a) and $2.5 \times 10^{-2}\omega_r$. The driving frequency is $\omega_d = 0.99\omega_r$, or $\Delta_r = 10^{-2}\omega_r$. $g = 5 \times 10^{-3}\omega_r$ and $\gamma = \Gamma = 1.5 \times 10^{-4}\omega_r$. The dashed lines show the resonance conditions that given by 1-photon, 2-photon and 3-photon process. Here $N_{\max} = 5$ and $m_{\max} = 4$ are used.

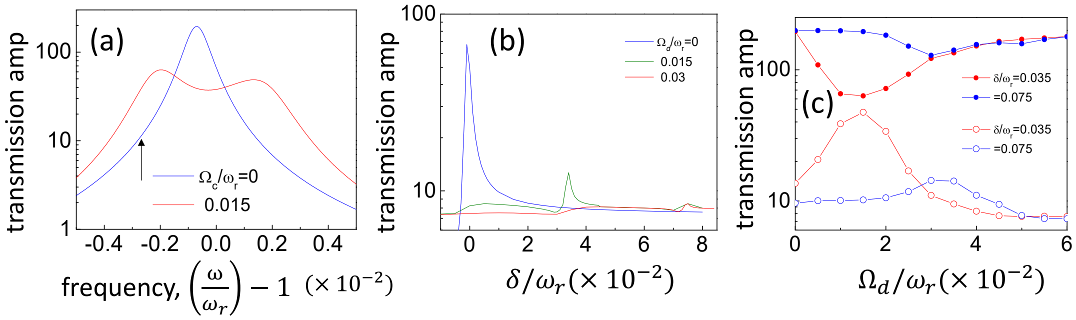

FIG. 3: (a) The log-scale transmission amplitude at $\delta/\omega_r = 3.5 \times 10^{-2}$ at the driving amplitude $\Omega_d/\omega_r = 0$ and 1.5×10^{-2}. The vertical arrow marks a good operation point for monitoring the amplitude change. (b) The transmission amplitude as a function of detuning δ/ω_r at a probing frequency slightly detuned from ω_r by a factor of 2.5×10^{-2} at $\Omega_d/\omega_r = 0$(blue), 1.5×10^{-2}(green) and 3×10^{-2}(red). There are resonance peaks at $\delta/\omega_r = 0$, 0.35 and 0.75 in each curve. (c) The peak height of transmission as a function of driving amplitude Ω_d at $\delta/\omega_r = 3.5 \times 10^{-2}$(red hollow circle) and 7.5×10^{-2}(blue hollow circle). The transmission amplitude of a probing frequency slightly detuned from ω_r by a factor of 2.5×10^{-2} at $\delta/\omega_r = 3.5 \times 10^{-2}$(red solid circle) and 7.5×10^{-2} (blue solid circle). The detuned measurement can be used for monitoring the peak heigh change.

a greatest change in transmission amplitude at $\Omega_d/\omega_r = 1.5 \times 10^{-2}$, featuring a large effective coupling strength g_{eff}. When g_{eff} is not larger than Γ, the resonator and dressed atom are only weakly coupled and the clear splitting is not observable. Though the transmission peak height gives a way to quantify g_{eff}, but it turns out not very practical to measure the peak height when the resonance is shifting. When the shift is small, we can apply the approach similar to the dispersive probe for a qubit readout by simply setting a detuned probing frequency, at which there is a large amplitude change due to frequency shift. As an example, we set a probing frequency marked by the vertical arrow in SFig. 3(a). SFig. 3(b) illustrates the transmission amplitude at the selected probe frequency as a function of δ/ω_r at $\Omega_d/\omega_r = 0$, 1.5×10^{-2} and 3×10^{-2}. One can clear see that each curve present the resonance conditions for $m = 1$, 2 and 3, respectively. By choosing these resonance conditions, and plot the transmission amplitude at the selected probe frequency as a function of driving amplitude, one could get the red and blue solid symbols in SFig. 3(c). The data agree well to the transmission peak height of the resonator plotted by red and blue hollow symbols, confirming an effective way of study g_{eff} in this system.

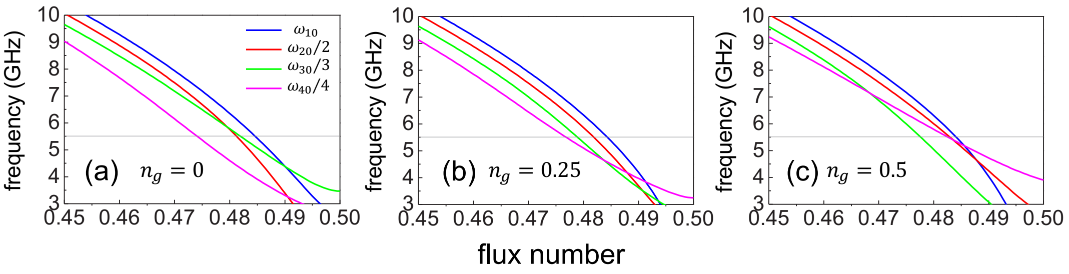

FIG. 4: The CPB/transmon energies ω_{m0}/m with m from 1 to 4 at $n_g = 0$(a), 0.25(b) and 0.5(c). The horizontal line is the probe frequency 5.514 GHz.

II. CALCULATIONS ON ENERGIES OF COOPER-PAIR BOXES AND TRANSMONS

The energies of Cooper-pair boxes and transmons is considered by using the Hamiltonian presented in the charge number basis $|n\rangle$.

$$H_{\text{CPB}} = 4E_C(n - n_g)^2|n\rangle\langle n| + \frac{E_J}{2}\sum_n |n+1\rangle\langle n| + |n-1\rangle\langle n|,$$

in which E_C and E_J are charging energy and Josephson energy, respectively. n_g is gate charge number. In our sample, E_J is tunable by the biased magnetic flux number f, $E_J = E_J(0)|\cos\pi f|$. In a small frequency range, we have determined how the atom energy changes as a function of f when $\omega_{10} = 5.5$ GHz. As such the transition energy ω_{20} as well as the anharmonicity $\alpha = \omega_{10} - \omega_{21} = 1$ GHz is determined. Although for the transmon $\alpha \sim E_C$, but it is not accurate in our case that $E_J/E_C \sim 10$. To find the accurate E_C value, we set $\omega_{10} = 5.5$ GHz, $\alpha = \omega_{10} - \omega_{21} = 1$ GHz, and $n_g = 0.25$ to find the reasonable $E_C = 0.65$ GHz and $E_J/E_C = 11.5$, which are not sensitive to n_g.

With the estimated flux number $f_r = 0.484$ when $\omega_{10} = 5.5$ GHz, we can estimate the zero-flux Josephson energy $E_J(0)/E_C \sim 235$ and $E_J(0) \sim 150$ GHz. The higher excited level energies with our low E_J/E_C system show greater n_g dependence. In explaining the high P_p 1-tone experiment, we calculated ω_{m0}/m with m from 1 to 4. As illustrated in SFIG. 4, only when $n_g \sim 0.25$ one can have resonances roughly equal spaced in flux number change as shown in the measurement data.

The transmon spectroscopy was measured by sweeping the bias flux at a fix probe frequency. In SFig. 5(a)-(c), we display the transmission amplitude change as a function of bias flux at 6.912 GHz(a), 5.212 GHz(b) and 4.250 GHz(c). When the transmon energy becomes the same as the probe microwave photon energy, the transmission amplitude reduces due to resonance absorption. The transmon frequency (ω_{10}) as a function of flux number can therefore be summarized as shown in SFig. 5(d), in which blue circles and red crosses are for 1st and 2nd cooldowns. The dressed state measurement was conducted in the 1st cooldown but transmon spectroscopy in a wider frequency range was not performed until the 2nd cooldown. There is some spread in the experimental result because of finite line width and instability in flux biasing. Nevertheless, we can determine that the $E_J(0)/E_C$ values are 235 and 170 in the 1st and 2nd cooldowns, respectively. The calculated ω_{10} is presented as solid and dash curves for 1st and 2nd cooldowns, respectively. Such a reduction in Josephson energy in our samples are common due to the continuous oxidation of Al junction when exposed to the air.

III. DERIVATION OF TRANSMISSION COEFFICIENT AT A DETUNED FREQUENCY

To derive the transmission coefficient for an atom coupled to a resonator, we apply the similar approach as in Sec. I by considering 2-level approximation for atom states as well as resonator states. The free hamiltonian of the composite system can expressed as

$$H = \omega_a \begin{pmatrix} 1 & 0 \\ 0 & 1 \end{pmatrix} \otimes \begin{pmatrix} 0 & 0 \\ 0 & 1 \end{pmatrix} + \omega_r \begin{pmatrix} 0 & 0 \\ 0 & 1 \end{pmatrix} \otimes \begin{pmatrix} 1 & 0 \\ 0 & 1 \end{pmatrix}.$$

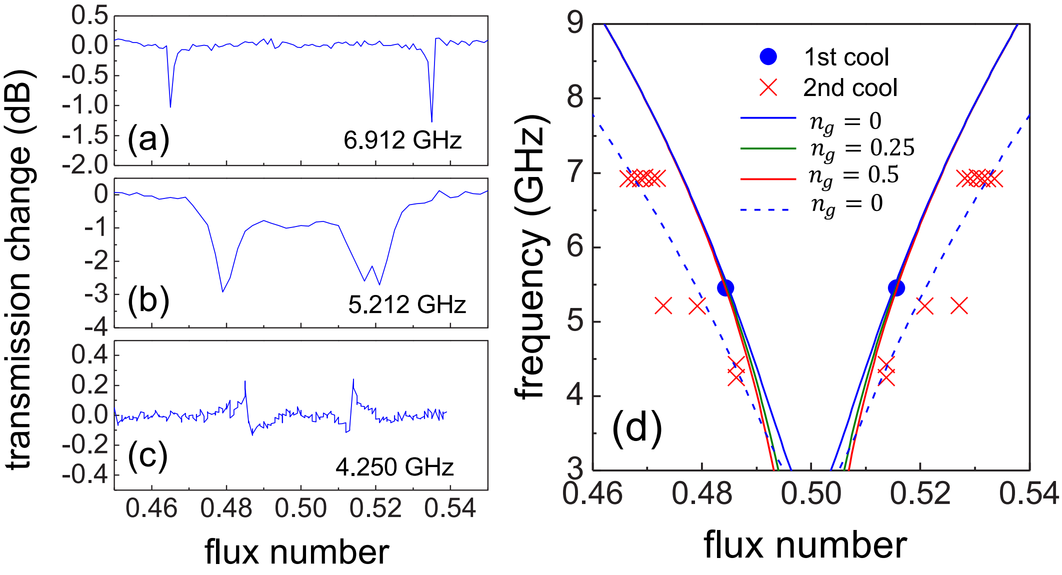

FIG. 5: (a)-(c) The transmission amplitude change(in unit of dB) as a function of bias flux for the transmon in the 2nd cooldown at the probe frequencies 6.912 GHz(a), 5.212 GHz(b) and 4.250 GHz(c). The sharp dips indicate the flux at which the transmon frequency is in resonance to the probe frequency. (d) The transmon frequency (ω_{10}) as a function of flux number obtained by the resonant absorption measurement. Blue circles and red crosses are for 1st and 2nd cooldowns. The solid curves are model calculation results for $n_g = 0$ (blue), 0.25(green) and 0.5 (red) for $E_J(0)/E_C = 235$. For the 2nd cooldown, a reduced Josephson coupling energy $E_J(0)/E_C = 170$ shown as the dashed curve($n_g = 0$) is better in fitting the measurement data.

The first and second 2×2 matrices note the operators for 2-level resonator and 2-level atom, respectively. $\omega_a = \omega_{10}$ is the level spacing. With the Jaynes-Cummings type interaction, the effective hamiltonian read as

$$H = \begin{pmatrix} 0 & 0 & 0 & 0 \\ 0 & \omega_a & g & 0 \\ 0 & g & \omega_r & 0 \\ 0 & 0 & 0 & \omega_a + \omega_r \end{pmatrix}.$$

When calculate the susceptibility, we need to calculate the correlation functions $\langle \Sigma_-(t)\Sigma_p(0)\rangle$ and $\langle \Sigma_p(0)\Sigma_-(t)\rangle$ of the destruction operators for the resonator.

$$\Sigma_- = \begin{pmatrix} 0 & 1 \\ 0 & 0 \end{pmatrix} \otimes \begin{pmatrix} 1 & 0 \\ 0 & 1 \end{pmatrix}, \qquad \Sigma_p = \begin{pmatrix} 0 & 1 \\ 1 & 0 \end{pmatrix} \otimes \begin{pmatrix} 1 & 0 \\ 0 & 1 \end{pmatrix}.$$

These correlation functions are

$$\langle \Sigma_-(t)\Sigma_p(0)\rangle = \rho_{p';20}(t) + \rho_{p';31}(t),$$

and

$$\langle \Sigma_p(0)\Sigma_-(t)\rangle = \rho_{p;20}(t) + \rho_{p;31}(t).$$

ρ_p and $\rho_{p'}$ are solutions to the master equation for ρ with the initial conditions of $\rho_s\Sigma_p$ and $\Sigma_p\rho_s$, respectively. Without loss of generality, ρ_s can be used as the ground state density matrix, and yield

$$\rho_p(0) = 0, \qquad \rho_{p'}(0) = \begin{pmatrix} 0 & 0 & 0 & 0 \\ 0 & 0 & 0 & 0 \\ 1 & 0 & 0 & 0 \\ 0 & 0 & 0 & 0 \end{pmatrix}.$$

The non-vanishing differential equations for $\rho_{p'}(0)$ are:

$$\frac{d}{dt}\begin{pmatrix} \rho_{p';10} \\ \rho_{p';20} \end{pmatrix} = -i \begin{pmatrix} \omega_a - i\gamma & g \\ g & \omega_r - i\Gamma \end{pmatrix} \begin{pmatrix} \rho_{p';10} \\ \rho_{p';20} \end{pmatrix},$$

with the initial condition

$$\begin{pmatrix} \rho_{p';10} \\ \rho_{p';20} \end{pmatrix} = \begin{pmatrix} 0 \\ 1 \end{pmatrix}.$$

Here γ and Γ are decay rates for atom and resonator, respectively. We could assume $\gamma = \Gamma$ for simplicity. By defining the parameter $\delta = \omega_a - \omega_r$, the solutions read

$$\rho_{p';10} = \cos\frac{\theta}{2}\sin\frac{\theta}{2}e^{-\gamma t}\left(e^{-i\Omega_- t} - e^{-i\Omega_+ t}\right) \tag{2}$$

$$\rho_{p';20} = \cos^2\frac{\theta}{2}e^{-\gamma t}e^{-i\Omega_- t} + \sin^2\frac{\theta}{2}e^{-\gamma t}e^{-i\Omega_+ t}, \tag{3}$$

in which $\cot\theta = \delta/2g$ and

$$\Omega_\pm = \omega_r + \frac{\delta}{2} \pm \sqrt{\frac{\delta^2}{4} + g^2}$$

The susceptibility is

$$\chi(\omega) = -\frac{\cos^2\frac{\theta}{2}}{\omega - \Omega_- + i\gamma} - \frac{\sin^2\frac{\theta}{2}}{\omega - \Omega_+ + i\gamma}.$$

By introducing a slightly detuned probe at $\omega = \omega_r + \Delta_p$ with $\Delta_p \ll \omega_r$, one can easily determine the transmission peak by maximizing the imaginary part of $\chi(\omega)$

$$Im\chi(\omega) = \frac{1}{\gamma}\frac{\cos^2\frac{\theta}{2}}{(\omega - \Omega_-)^2 + \gamma^2} + \frac{1}{\gamma}\frac{\sin^2\frac{\theta}{2}}{(\omega - \Omega_+)^2 + \gamma^2}$$

by varying δ. We note that the real part is around zero at resonance. The maximum transmission amplitude would occur at

$$\delta = \Delta_p - \frac{g^2 + \gamma^2}{\Delta_p}.$$

If $\gamma \ll \Delta_p \sim g$, the associated peak height is majorly contributed from the imaginary part of $\chi(\omega)$:

$$h \propto \frac{1}{\gamma}\frac{g^2 + \gamma^2}{\Delta_p^2 + g^2 + \gamma^2},$$

as stated in maintext Eq. (4).

---

[1] A. F. Kockum, M. Sandberg, M. R. Vissers, J. Gao, G. Johansson, and D. P. Pappas, Journal of Physics B: Atomic, Molecular and Optical Physics **46**, 224014 (2013).
